# RGMa inhibition with human monoclonal antibodies promotes regeneration, plasticity and repair, and attenuates neuropathic pain after spinal cord injury

**DOI:** 10.1038/s41598-017-10987-7

**Published:** 2017-09-05

**Authors:** Andrea J. Mothe, Nardos G. Tassew, Alirezha P. Shabanzadeh, Romeo Penheiro, Robin J. Vigouroux, Lili Huang, Christine Grinnell, Yi-Fang Cui, Emma Fung, Philippe P. Monnier, Bernhard K. Mueller, Charles H. Tator

**Affiliations:** 1 Krembil Research Institute, Division of Genetics and Development, Krembil Discovery Tower, Toronto ON, M5T 2S8 Canada; 2 Toronto Western Hospital, University Health Network, Toronto ON, M5T 2S8 Canada; 3 AbbVie Bioresearch Center, Worcester MA, 01605 USA; 4 Neuroscience Research, Research and Development, AbbVie Deutschland GmbH & Co. KG, Knollstrasse, Ludwigshafen, 67061 Germany; 5 Department of Ophthalmology and Vision Science, University of Toronto, Toronto ON, M5S 3H6 Canada; 6 Department of Surgery, Division of Neurosurgery, University of Toronto, Toronto ON, M5S 3H6 Canada

## Abstract

Traumatic spinal cord injury (SCI) causes a cascade of degenerative events including cell death, axonal damage, and the upregulation of inhibitory molecules which prevent regeneration and limit recovery. Repulsive guidance molecule A (RGMa) is a potent neurite growth inhibitor in the central nervous system, exerting its repulsive activity by binding the Neogenin receptor. Here, we show for the first time that inhibitory RGMa is markedly upregulated in multiple cell types after clinically relevant impact-compression SCI in rats, and importantly, also in the injured human spinal cord. To neutralize inhibitory RGMa, clinically relevant human monoclonal antibodies were systemically administered after acute SCI, and were detected in serum, cerebrospinal fluid, and in the injured tissue. Rats treated with RGMa blocking antibodies showed significantly improved recovery of motor function and gait. Furthermore, RGMa blocking antibodies promoted neuronal survival, and enhanced the plasticity of descending serotonergic pathways and corticospinal tract axonal regeneration. RGMa antibody also attenuated neuropathic pain responses, which was associated with fewer activated microglia and reduced CGRP expression in the dorsal horn caudal to the lesion. These results show the therapeutic potential of the first human RGMa antibody for SCI and uncovers a new role for the RGMa/Neogenin pathway on neuropathic pain.

## Introduction

Spinal cord injury (SCI) is a devastating condition with great personal and societal costs. Despite advances in clinical care, currently there is no effective treatment to enhance regeneration after major SCI. Following the initial trauma, there is a cascade of molecular and degenerative events including apoptosis, ischemia, excitotoxicity, and the upregulation of inhibitory molecules^1^. Neuronal death and inhibition of axonal regeneration limit neurological recovery following injury. CNS axons show a limited capacity to regenerate and often retract away from the injury site or undergo secondary axonal degeneration due to intrinsic mechanisms and the inhibitory environment of the injured spinal cord. Early studies demonstrating that adult CNS axons can regenerate through a peripheral nerve graft^2^ suggested that the local environment of the adult CNS is a major cause for the lack of regeneration. Extracellular inhibitory proteins such as Nogo-A, myelin associated glycoprotein, and oligodendrocyte myelin glycoprotein are present in CNS myelin and combined with the accumulation of chondroitin sulfate proteoglycans (CSPG), comprise the inhibitory glial scar that forms after SCI. Neutralization of these inhibitory proteins by antibodies^3, 4^ or enzymatic treatment to reduce CSPG-induced inhibition^5–7^ have shown partial improvements. Thus, a better characterization of inhibitory molecules in the injured CNS and strategies that reduce inhibition are of great interest.

Repulsive Guidance Molecule A (RGMa)^8^ is a GPI-linked glycoprotein that exists in membrane-bound and soluble forms, both of which inhibit neurite growth^9^ by binding to its neuronal receptor, Neogenin^10^. Notably, RGMa is present in both the glial scar and myelin and accumulates in lesioned areas after traumatic injury of the brain and spinal cord in rodent models^11–14^. RGMa is also present in multiple sclerosis patients’ active and chronic lesions and in amyloid plaques of patients diagnosed with Alzheimer’s disease^14, 15^. Neutralization of RGMa with rat antibodies recognizing the C-terminal portion of RGMa promoted axonal growth in rats with a thoracic dorsal hemisection spinal cord lesion^13^.

The RGMa receptor, Neogenin, requires localization to lipid raft domains in the plasma membrane to either transmit guidance signals or control cell death^16^. Lipid rafts are microdomains within the plasma membrane of cells which are enriched with protein receptors important for cell signalling^17^. Lipid rafts have been implicated in regulating the activity of various guidance receptors, such as the Netrin-1 receptor Deleted in Colorectal Cancer^18^, and allows neurons to respond to cues such as Brain Derived Neurotrophic Factor or Semaphorin^19^. Using a peptide strategy to prevent Neogenin translocation to lipid rafts, we could promote neuroprotection and axonal regeneration after rat SCI^16^.

Toward the goal of clinical translation, we developed clinically relevant human anti-RGMa monoclonal antibodies that also prevent Neogenin association with lipid rafts. These human monoclonal antibodies (mabs) are selective for the N-terminal portion of RGMa which act similarly to both neutralize RGMa and inhibit Neogenin function^14^. A limitation to clinical application of antibodies is the route of administration as intrathecal delivery may induce secondary damage, thus there is great interest in developing strategies that allow systemic treatment of SCI. Here, we characterized RGMa expression after impact-compression injury and we examined the efficacy of inhibiting RGMa by systemic administration of two different human anti-RGMa antibodies in a clinically relevant model of acute SCI.

## Results

### Inhibitory RGMa is upregulated following rat and human spinal cord injury

**Figure 1:**
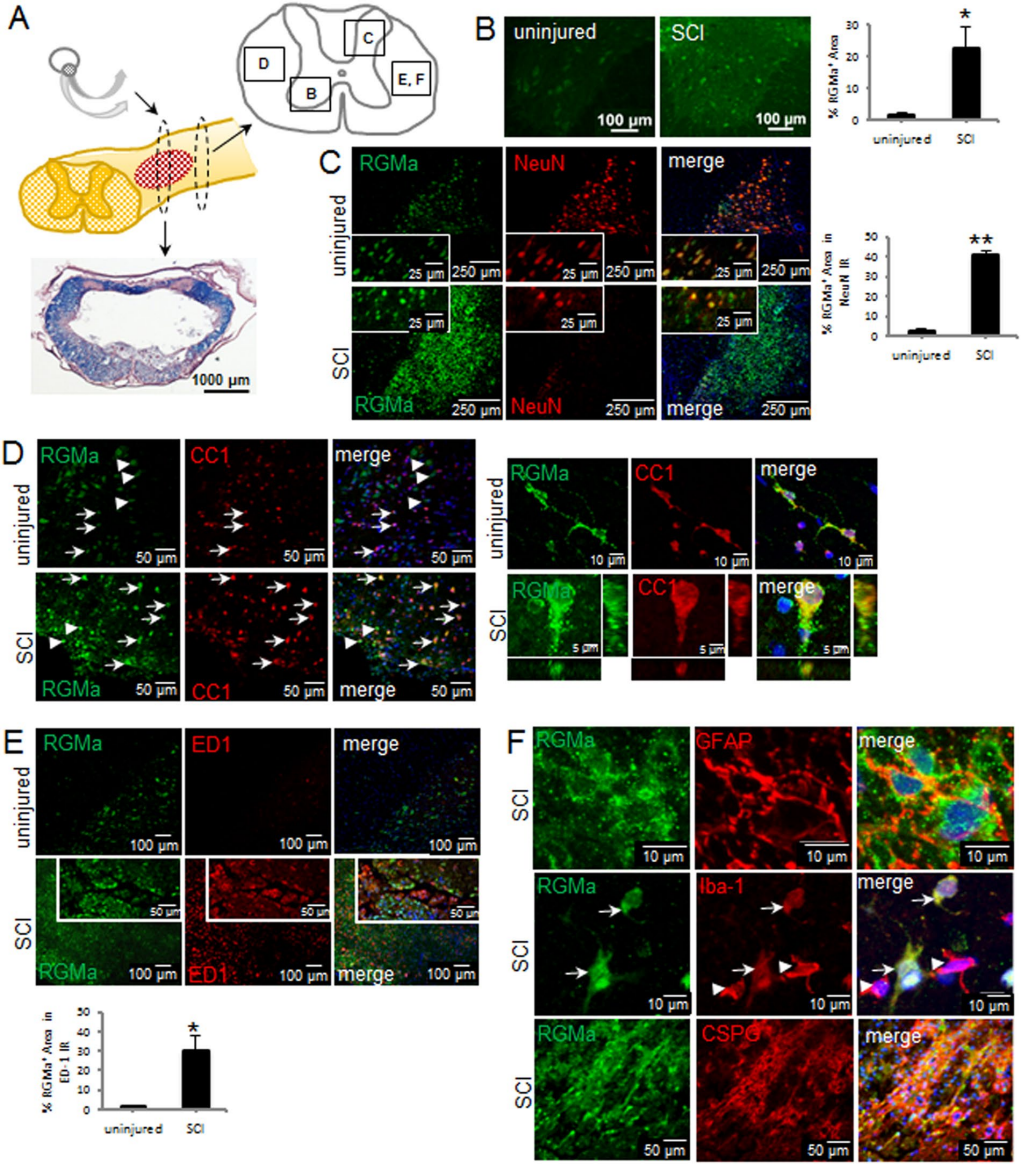
RGMa is upregulated in the adult rat spinal cord after injury. (**A**) Schematic diagram illustrates the extradural impact-compression spinal cord injury produced with a modified aneurysm clip. The red hatched area depicts the lesion site. The lesion epicentre demonstrates central cavitation with a spared rim of white matter as shown in the LFB/H&E stained section. Schematic diagram depicts regions (boxed areas) of the spinal cord that were imaged in the uninjured cord and in the injured cord immediately caudal to the lesion site. (**B**) RGMa immunostaining in the ventral horn gray matter in uninjured rat spinal cord and at 1 week post-SCI. Quantification of % RGMa+ area showed significant upregulation after SCI. Data are mean ± SEM (uninjured, n = 3; SCI, n = 4); *p < 0.05 (Student’s t-test). (**C**) In the uninjured rat spinal cord, RGMa was primarily expressed by neurons. Insets show higher magnification of RGMa (green) and NeuN (red) double-labeled cells. After injury, perilesional neurons showed increased expression of RGMa (shown 1 week post-SCI). The same regions of the dorsal horn (box C in Fig. 1A) were imaged in transverse sections of the uninjured and injured cord. Quantification of % RGMa+ area in the NeuN immunoreactive (IR) regions showed significant upregulation after SCI. Data are mean ± SEM (uninjured, n = 3; SCI, n = 4); **p < 0.01 (Student’s t-test). (**D**) In the uninjured and injured rat spinal cord, oligodendrocytes (CC1, red) expressed RGMa (green). RGMa+ /CC1 + double-labeled cells are indicated by arrows, and RGMa+ /CC1− cells are indicated by the arrowheads. Comparable regions of the white matter (box D in Fig. 1A) were imaged in the uninjured and injured spinal cord. High magnification confocal Z-stack images of an oligodendrocyte in spared gray matter of the injured spinal cord showing RGMa+/CC1+ double-labeling in the XZ and YZ orthogonal planes. Merged panels show DAPI nuclear counterstaining. Quantification of % RGMa+ area in the CC1 immunoreactive (IR) regions showed significant upregulation of RGMa after SCI. Data are mean ±SEM (uninjured, n = 3; SCI, n = 4); *p < 0.05 (Student’s t-test). (**E**) RGMa expression was apparent in ED-1 + regions (box E in Fig. 1A) after SCI. Insets show higher magnification of RGMa (green) and ED-1 (red) double-labeled macrophages. The uninjured spinal cord showed no ED-1 immunoreactivity. Quantification of % RGMa+ area in the ED-1 IR regions (box E) showed significant upregulation of RGMa after SCI. Data are mean ±  SEM (uninjured, n = 3; SCI, n = 4); *p < 0.05 (Student’s t-test). (**F**) After SCI, RGMa was expressed by astrocytes (GFAP), and within CSPG scar-rich regions within and surrounding the lesion site. Activated microglia and macrophages also expressed RGMa (Iba-1 + microglia shown). RGMa+ /Iba-1 + double-labeled cells are indicated by arrows, and RGMa-/Iba-1 + cells are indicated by the arrowheads. Merged panels show DAPI nuclear counterstain.

The most common form of SCI in humans is impact-compression, however it was unknown whether RGMa is upregulated after this type of injury as was shown after hemisection SCI in the rat^11, 13^. Here, we showed that RGMa is markedly upregulated after clip impact-compression injury of the rat spinal cord, which is a clinically relevant model of SCI reflecting human pathology. The bilateral impact-compression model of SCI we used demonstrates central cavitation with a spared rim of white matter tissue, as shown in Fig. 1A. At 1 week after SCI, there was a significant 15-fold increase in RGMa expression (Fig. 1B). As shown by double-label immunostaining, RGMa was primarily expressed by neurons (RGMa+/NeuN+) and oligodendrocytes (RGMa+/CC1+) in the normal uninjured rat spinal cord, and after impact-compression injury, RGMa expression was significantly upregulated (Fig. 1C). After SCI, RGMa was expressed in neurons (Fig. 1C), oligodendrocytes (Fig. 1D), astrocytes as shown by GFAP labeling (Fig. 1F), activated microglia and macrophages as shown by Iba-1 (Fig. 1F) and ED-1 (Fig. 1E), and within CSPG scar-rich regions within and surrounding the lesion site (Fig. 1F). CSPG expression is low in the normal adult spinal cord, coincident with lower levels of RGMa. In the uninjured spinal cord, there was also low RGMa expression in Iba-1+ microglia, and very little RGMa staining apparent in GFAP+cells (data not shown). Quantification of the upregulation of RGMa expression was determined in sections double-labeled for RGMa and cell-type specific markers (Fig. 1C–E).

**Figure 2:**
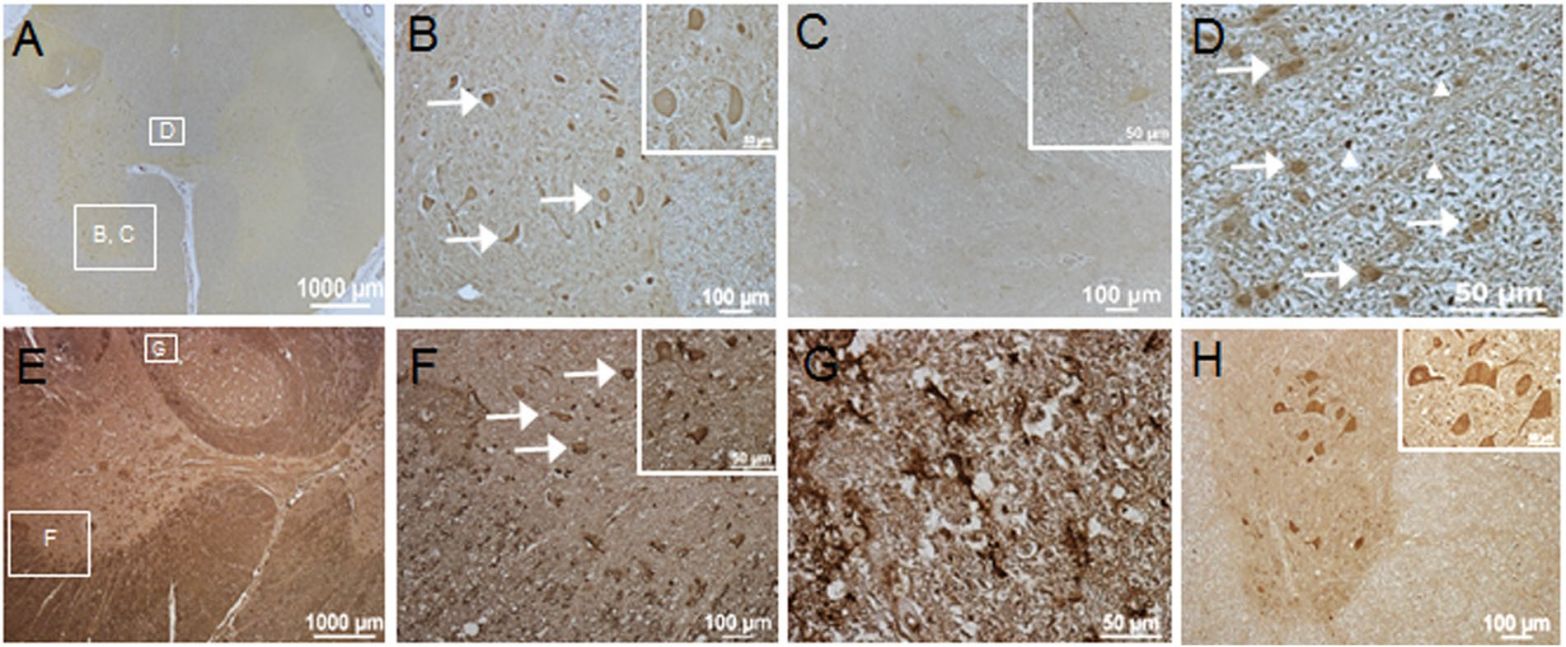
RGMa is expressed in the adult human spinal cord and upregulated after injury. (**A**–**D**) Cross-sections of uninjured human spinal cord (obtained from a 52 year old female donor) showing low levels of RGMa. (**A**) Low magnification image showing low levels of RGMa. (**B**) Higher magnification of boxed region (box labeled B in panel A) showing RGMa expression in neurons (arrows) in the anterior horn (inset shows higher magnification of RGMa+ neurons). (**C**) Adjacent section stained with RGMa antibody pre-absorbed with RGMa peptide showing specificity of staining (inset shows higher magnification). (**D**) Higher magnification of boxed region (box labeled D in panel A) showing RGMa immunoreactivity in the dorsal column (arrows, glia; arrowheads, axons). (**E**–**G**) Cross-sections of injured human spinal cord (3d post-injury from an 84 year old male patient) showing upregulation of RGMa. (**E**) Low magnification section caudal to lesion site showing higher levels of RGMa expression. (**F**) Higher magnification of boxed region (box labeled F in panel E) showing RGMa expression in spared neurons (arrows) in the anterior horn (inset shows higher magnification). (**G**) Higher magnification of boxed region (box labeled G in panel E) showing RGMa immunoreactivity in axons and glia in the dorsal column. (**H**) Expression of the RGMa receptor, Neogenin, in anterior horn neurons in uninjured human spinal cord.

We then asked whether RGMa is similarly expressed in the adult human spinal cord after injury. For the first time, we demonstrate RGMa expression in the human spinal cord and its upregulation after injury. In the uninjured human spinal cord, RGMa was expressed at low levels in neurons, as shown by RGMa immunostaining of neurons identified via characteristic neuronal morphology in the intermediate gray matter (Fig. 2A,B) and glial cells in the dorsal column (Fig. 2D). In the injured human spinal cord at 3 days post-SCI, RGMa expression was significantly upregulated in neurons, axons, glial cells, and myelin enriched white matter regions (Fig. 2E–G). Furthermore, the RGMa receptor Neogenin, was expressed by neurons in both rat (data not shown) and human spinal cord (Fig. 2H).

### Human anti-RGMa mabs promote neurite outgrowth *in vitro*

**Figure 3:**
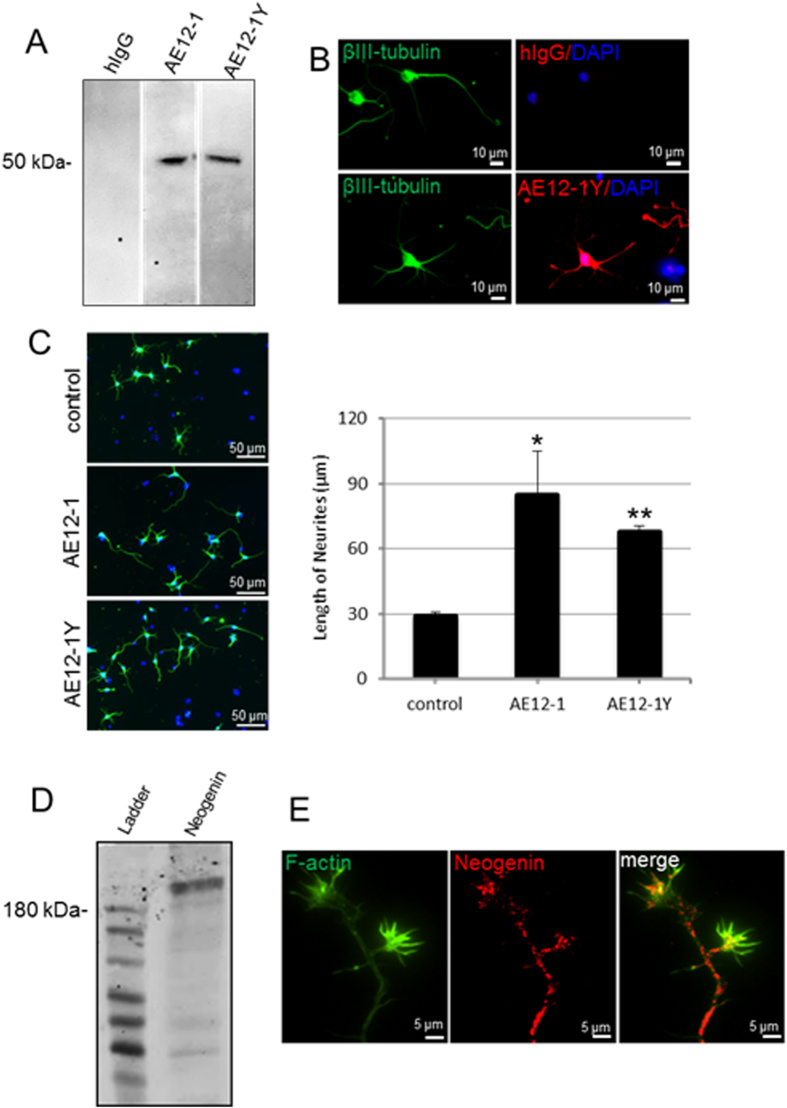
Human mabs recognize and neutralize RGMa. (**A**) Human mabs AE12-1 and AE12-1Y recognized a 50 kDa band on Western blots of mouse cortical neuron lysates. No bands were apparent with the hIgG isotype control antibody. Full-length blots are presented in Supplementary Figure 1. (**B**) Cultured mouse primary cortical neurons (immunostained with βIII-tubulin, green) expressed RGMa (red) as shown by AE12-1Y immunostaining. No staining was apparent with the hIgG isotype control. (**C**) Axonal outgrowth assay. E18 mouse cortical neurons plated on laminin and inhibitory RGMa showed minimal extension of neurites in controls incubated with hIgG. In contrast, incubation with AE12-1 and AE12-1Y RGMa antibodies resulted in more extensive neurite extension (βIII-tubulin, green; DAPI, blue). Neutralization of RGMa with AE12-1 and AE12-1Y significantly increased cortical neurite outgrowth. Experiments performed in triplicate. Data are mean ± SEM; *p < 0.05; **p < 0.01 (t-test compared to control). (**D**,**E**) Expression of the RGMa receptor Neogenin. (**D**) Western blot of adult rat brain lysates showing Neogenin expression. (**E**) Cultured mouse cortical neurons (3 days *in vitro*; F-actin, green) expressed Neogenin (red).

Previous experiments showing that RGMa neutralization promotes functional recovery after hemisection SCI^13^ were performed using rabbit polyclonal antibodies. Hence, the development of a clinically relevant monoclonal antibody for human RGMa that promotes regeneration has become the target of intense investigation. The human antibodies AE12-1 and AE12-1Y are high-affinity RGMa-selective mabs with comparable binding to human, rat and mouse RGMa. The 2 mabs differ in their half-life, with AE12-1 having a longer half-life of 6 days compared to AE12-1Y half-life of 2 days. As shown previously, the monoclonal antibodies are specific for RGMa^20^. In Western blots of mouse cortical neuron lysates, both mabs are specific for RGMa and no cross-reactivity was observed between other RGM members (Fig. 3A). Cultured mouse primary cortical neurons also expressed RGMa, as shown by immunostaining with the AE12-1Y mab (or AE12-1, immunostaining not shown) (Fig. 3B). We then showed that the anti-RGMa antibodies promote neurite outgrowth *in vitro*. Cultured embryonic mouse cortical neurons plated on laminin and inhibitory RGMa showed minimal extension of neurites when incubated with human IgG (hIgG) isotype control whereas incubation with AE12-1 and AE12-1Y RGMa antibodies resulted in significantly more extensive neurite outgrowth (Fig. 3C). In Western blots, the Neogenin receptor was detected as a 200-kDa band (Fig. 3D) and Neogenin was also expressed by cultured mouse cortical neurons (Fig. 3E).

### RGMa human antibodies are detected in rat serum, CSF, and injured spinal cord

**Figure 4:**
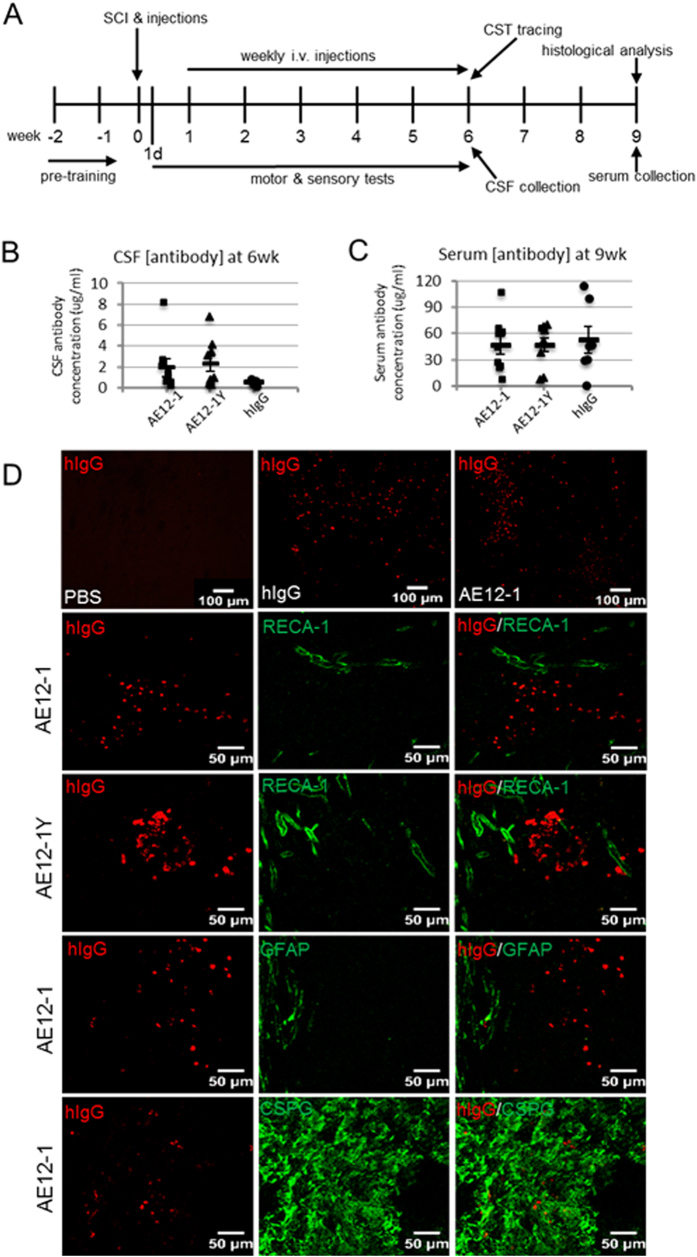
Study design, antibody concentration in CSF and serum, and detection of antibodies in spinal cord tissue. (**A**) Summary of experimental timeline. Rats were pre-trained on the ladderwalk and CatWalk prior to SCI. The mabs AE12-1 or AE12-1Y or controls (hIgG or PBS vehicle) were injected immediately after impact-compression SCI. Rats were tested for the BBB locomotor score and motor subscore at 1 day post-SCI and then weekly. Rats were tested weekly on the ladderwalk when they were able to cross the horizontal ladder. Gait parameters were assessed using CatWalk at 6 weeks post-SCI, and sensory testing was conducted at 2 and 6 weeks post-SCI. At 6 weeks post-SCI, cerebrospinal fluid (CSF) was sampled via lumbar puncture and BDA was injected into the sensorimotor cortex for anterograde labeling of the corticospinal tract (CST). At 9 weeks post-SCI, serum was collected and rats were perfused for histological analysis. Using an ELISA assay, the concentration of human IgG in AE12-1 and AE12-1Y and hIgG treated rats was measured in (**B**) CSF sampled at 6 weeks post-SCI (AE12-1, n = 9; AE12-1Y, n = 9; hIgG, n = 6), and in (**C**) serum obtained at endpoint at 9 weeks post-SCI (AE12-1, n = 9; AE12-1Y, n = 9; hIgG, n = 7). (**D**) Immunostaining of rat spinal cord tissue with anti-human IgG (red). No immunoreactivity for hIgG was apparent in rats injected with PBS. Immunoreactivity for hIgG was detectable around the lesion site in rats injected with hIgG, AE12-1, and AE12-1Y, as shown in the low magnification images in the upper panels. Higher magnification images are shown in the other panels. Human IgG (red) was detected around blood vessels as shown with RECA-1 (green) (tissue from rats injected with AE12-1 and AE12-1Y shown in panels, also same for hIgG) and within scar tissue (CSPG, green) (AE12-1 shown, also same for AE12-1Y and hIgG).

Factors limiting potential treatments for SCI include mode of administration of the therapeutic. As intrathecal administration via osmotic pumps and catheters may result in secondary tissue damage or associated problems such as catheter occlusion, we performed systemic administration of the RGMa mabs. A summary of the study design is depicted in Fig. 4A. Rats were pre-trained and then clip impact-compression SCI was made at T8, immediately followed by local intraspinal and intravenous (i.v.) injections of the mabs or controls (hIgG isotype control or PBS (phosphate buffered saline) vehicle). The i.v. injections were repeated weekly for 6 weeks. At 6 weeks post-SCI, cerebrospinal fluid (CSF) was sampled via lumbar puncture and the tracer biotinylated dextan amine (BDA) was injected into the sensorimotor cortex for anterograde labeling of the corticospinal tract (CST). At 9 weeks post-SCI, 3 weeks after the last antibody dose, serum was collected and rats were perfused. Using a ligand-based assay, we determined the concentration of human antibody in CSF and serum samples from AE12-1, AE12-1Y and hIgG treated rats. The antibody concentration in the CSF of rats injected with AE12-1 ranged from 0.25–8.20 ug/ml, and for AE12-1Y the antibody concentration range was 0.33–6.77 ug/ml, and for hIgG the range was 0.16–0.77 ug/ml (Fig. 4B). In comparison, antibody concentration in serum was considerably higher, approximately 10-fold greater than in CSF as the antibodies were injected intravenously (Fig. 4C). Furthermore, antibody concentration remained elevated in serum 3 weeks after the last dose. We also showed that the human antibodies were detected in the injured rat spinal cord by immunostaining of rat spinal cord tissue with anti-human IgG. Human IgG immunoreactivity was detected in tissue from rats injected with AE12-1, AE12-1Y, or hIgG control antibody but not in PBS vehicle controls (Fig. 4D). Staining of human antibody was apparent around blood vessels and within CSPG+scar tissue around the lesion site (Fig. 4D). Importantly, this suggests that RGMa mabs penetrated CNS tissues to neutralize RGMa.

### RGMa mabs improve functional recovery after SCI

**Figure 5:**
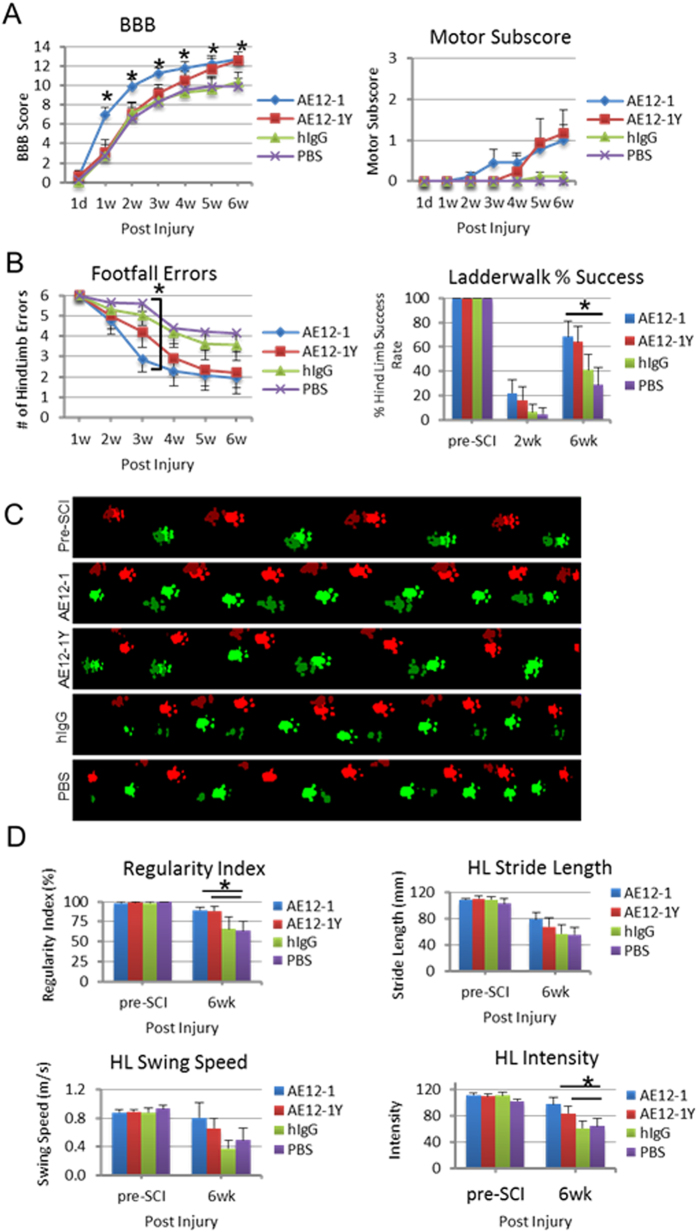
RGMa inhibition with human monoclonal antibodies promotes functional recovery after SCI. (**A**) Rats treated with mab AE12-1 showed significant improvement in BBB relative to hIgG and PBS controls at 1–6 weeks post-SCI. AE12-1Y treated rats showed significant improvement at 6 weeks post-SCI relative to controls. AE12-1 and AE12-1Y treated rats showed higher motor subscores relative to controls but this was not statistically significant. Data are mean ± SEM (AE12-1, n = 9; AE12-1Y, n = 9; hIgG, n = 9; PBS, n = 8); *p < 0.05, two-way repeated-measures ANOVA with Bonferroni’s post-hoc test. (**B**) Rats treated with AE12-1 showed fewer hindlimb footfall errors on the ladderwalk compared to PBS controls at 3 weeks post-SCI (*p < 0.05) and a trend towards reduced errors at 6 weeks (p = 0.07). At 6 weeks post-SCI, AE12-1 treated rats showed a higher percentage of successful hindlimb steps compared to control. Data are mean ± SEM (AE12-1, n = 9; AE12-1Y, n = 9; hIgG, n = 9; PBS, n = 8); two-way repeated-measures ANOVA, Bonferroni’s post-hoc test. (**C**) Representative footprints obtained from CatWalk gait analysis from pre-SCI and from each group at 6 weeks post-SCI. Forelimb paw prints are brighter than the hindlimb paw prints. (**D**) Rats treated with both mabs showed significant improvement in the regularity index relative to control groups. The mab treated rats showed a trend towards improved hindlimb stride length and swing speed. Rats injected with AE12-1 showed significantly higher hindlimb intensity values relative to controls. Data are mean ± SEM (AE12-1, n = 8; AE12-1Y, n = 8; hIgG, n = 8; PBS, n = 7); *p < 0.05, two-way ANOVA, Sidak’s multiple comparisons.

We then examined the effect of inhibiting RGMa with AE12-1 and AE12-1Y after acute impact-compression SCI. Neurological recovery was monitored weekly using the BBB locomotor rating scale, motor subscore, and horizontal ladderwalk test. Acute treatment with AE12-1 showed significant recovery of the BBB as early as 1 week post-SCI compared to PBS or hIgG controls which was maintained for the duration of the study (Fig. 5A). In contrast, AE12-1Y showed a delayed improvement on the BBB with a statistically significant difference at 6 weeks post-SCI relative to controls (12.6 vs 9.9 PBS). The difference in recovery profiles of AE12-1 and AE12-1Y may be due to the longer half-life of AE12-1. Both mabs also showed a trend towards a higher motor subscore compared to controls (Fig. 5A). We also assessed hindlimb coordination with the ladderwalk test in which footfall errors are scored, a higher score reflecting poorer coordination. The ladderwalk test showed a trend toward reduced footfall errors in rats treated with AE12-1 or AE12-1Y, with a statistically significant difference at 3 weeks post-SCI for AE12-1 (p < 0.05) and a trend towards reduced errors at weeks 4, 5, and 6 (Fig. 5B). At 6 weeks post-SCI, both AE12-1 and AE12-1Y treated rats showed a higher percentage of successful hindlimb steps (68.4% and 64.2%) compared to controls (hIgG 41%, PBS 29%). To further characterize the effects of RGMa neutralization on neurobehavioral function, we performed gait analysis using the CatWalk system at 6 weeks post-SCI and treatment (Fig. 5C). Rats treated with both mabs showed significant improvement in the regularity index relative to control groups, reflecting better inter-paw coordination (AE12-1 89.3%, AE12-1Y 88.3%, hIgG 65.8%, PBS 63.4%) (Fig. 5D). The mab treated rats also showed a trend towards improved hindlimb stride length and swing speed approaching the pre-SCI values, although this did not reach statistical significance. Interestingly, the mean intensity with which the hindpaws contacted the glass walkway significantly increased in AE12-1 treated rats compared to controls (Fig. 5D). The intensity threshold has previously been used to assess mechanical allodynia in a model of chronic neuropathic pain^21^. In addition, treatment with the human mabs did not alter rat weight (data not shown). Thus, this is the first study to show functional recovery after SCI and systemic injection of RGMa antibodies.

### RGMa mabs enhance neuronal survival

**Figure 6:**
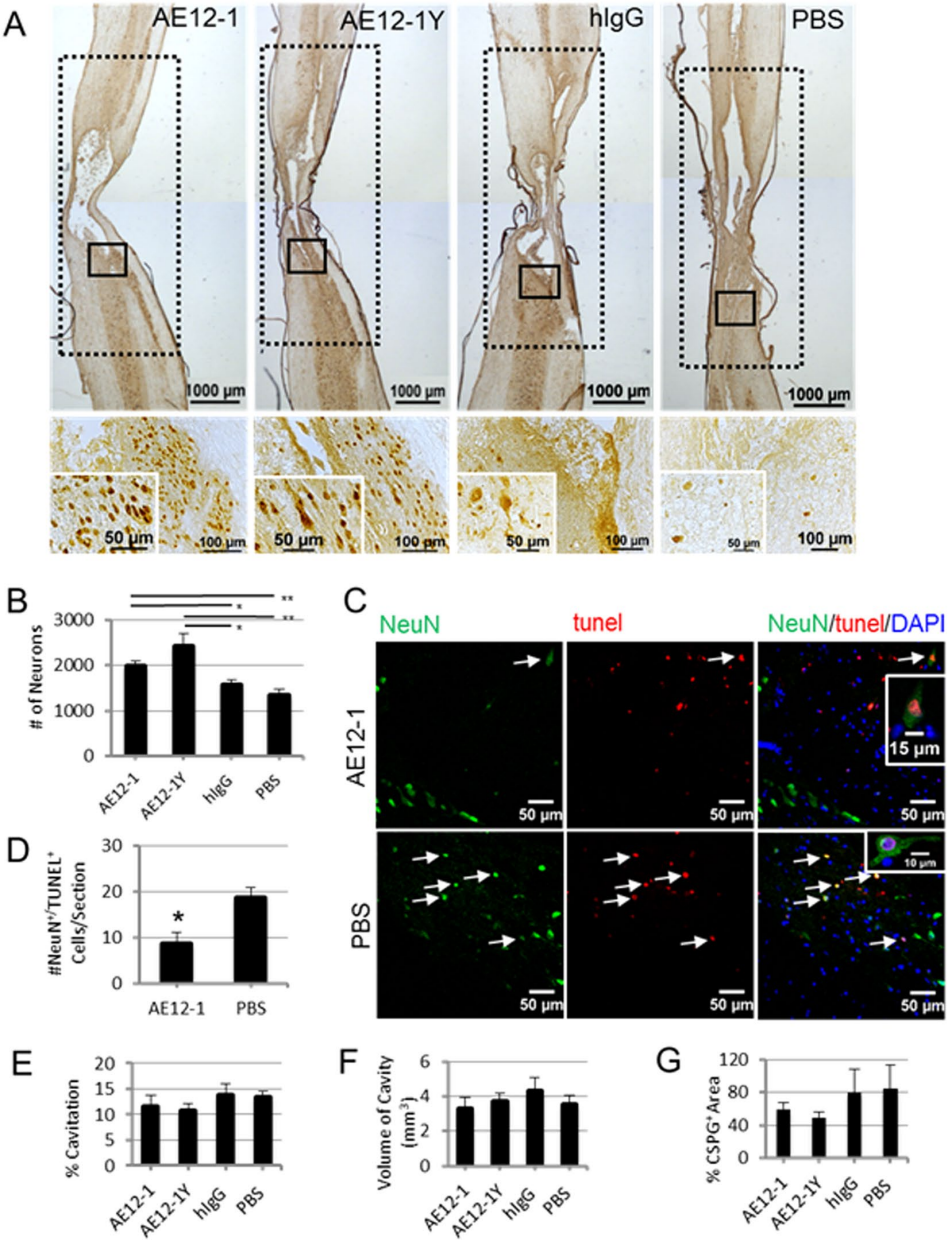
RGMa inhibition promotes neuronal survival by attenuation of apoptosis. (**A**) Low magnification of parasagittal sections of injured spinal cord 9 weeks post-SCI immunostained with the neuronal marker NeuN. Solid line boxed regions are magnified in bottom panels which contain insets showing neurons at high magnification. NeuN + neurons within regions outlined with dotted lines shown in low magnification images were quantitated. (**B**) Rats administered mabs AE12-1 and AE12-1Y showed significantly higher perilesional neuronal sparing than rats that received hIgG and PBS. Data are mean ± SEM (AE12-1, n = 7; AE12-1Y, n = 8; hIgG, n = 6; PBS, n = 7); *p < 0.05, **p < 0.01, one-way ANOVA with Bonferroni’s post-hoc test. (**C**) In a separate experiment, apoptosis of neurons was examined at 7 hour post-SCI from rats administered AE12-1 or PBS. Double-labeling with NeuN (green) and TUNEL (red) identified apoptotic neurons (arrows) in gray matter adjaent to the lesion. (**D**) The average number of NeuN+/TUNEL+ cells counted per section was significantly less in AE12-1 treated rats than in rats administered PBS vehicle. Data are mean ± SEM (AE12-1, n = 5; PBS, n = 3); *p < 0.05, t-test. (**E**) There was no significant difference in the percentage cavitation or (**F**) the volume of cavity between groups. (**G**) AE12-1 and AE12-1Y treated rats showed a trend towards reduced % CSPG + area at the lesion site. Data are mean ± SEM (AE12-1, n = 8; AE12-1Y, n = 8; hIgG, n = 6; PBS, n = 7).

In a rat model of optic nerve injury^22^, inhibiting the function of the Neogenin receptor promoted neuronal survival. Previously we showed that inhibiting Neogenin function with a peptide approach enhanced perilesional neuronal sparing^16^. Membrane fractionation experiments also demonstrated that injection of AE12-1Y altered the localization of Neogenin to the heavy membrane fraction, thus preventing the association of Neogenin with lipid rafts and blocking cell death signaling^14^. Therefore, we examined if neutralizing RGMa with the monoclonal antibodies could attenuate neuronal loss after SCI. After 6 weeks of AE12-1 or AE12-1Y treatment, perilesional neurons were quantified with the neuronal marker NeuN at 9 weeks post-SCI. Treatment with the RGMa antibodies increased the number of perilesional neurons approximately 1.5-fold compared to controls (Fig. 6A and B). To determine if the neuronal sparing was due to fewer neurons undergoing apoptosis after injury, rats were treated as before with acute injection of AE12-1. Double-labeling with NeuN and TUNEL staining was performed at 7 hours post-SCI, a time point when neurons have been shown to undergo apoptosis after injury^23^. There were significantly fewer NeuN+/TUNEL+ neurons in AE12-1 treated rats relative to control (2-fold difference) (Fig. 6C and D).

We then examined whether mab treatment affected the lesion size after SCI, but there was no significant difference between groups in either the percentage cavitation or the volume of the cavity (Fig. 6E,F). We also quantified the area of CSPG immunoreactivity around the lesion site and found a trend towards reduced CSPG expression in mab treated rats (Fig. 6G).

### Treatment with anti-RGMa mabs promote axonal regeneration

**Figure 7:**
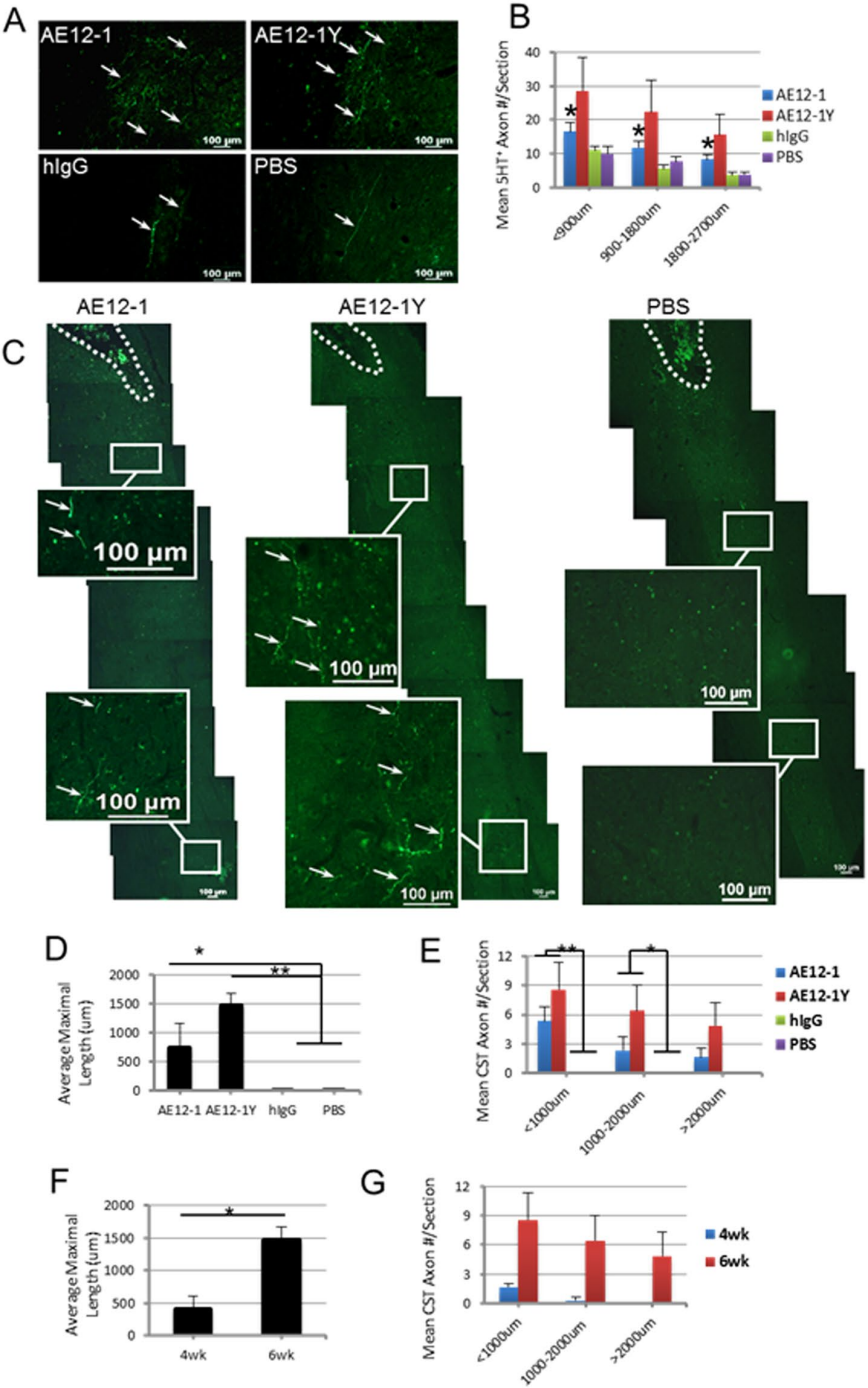
Treatment with anti-RGMa human mabs promotes axonal regeneration in the injured spinal cord. (**A**) 5HT immunoreactive serotonergic fibers (arrows) caudal to the lesion. (**B**) Quantification of the mean number of 5HT+ axons caudal to the lesion binned into progressive distances caudally. A significantly higher number of 5HT+ fibers were apparent in AE12-1 treated rats. Data are mean ± SEM (AE12-1, n = 8; AE12-1Y, n = 8; hIgG, n = 6; PBS, n = 6); *p < 0.05, one-way ANOVA with Bonferroni’s post-hoc test. (**C**) To visualize axons from the CST, BDA was injected at 6 weeks after SCI, and CST tracing analysis was performed at 9 weeks after SCI. Low magnification images of spinal cord. Rostral is at the top and the lesion cavity is outlined, and boxed regions are shown at higher magnification in the insets. Tracing with BDA revealed labeled fibers (arrows) caudal to the cavity in AE12-1 and AE12-1Y treated rats, but not in controls (PBS control shown). BDA+ fibers showed an irregular tortuous morphology. (**D**,**E**) Average maximal length of BDA labeled CST fibers increased after AE12-1 and AE12-1Y treatments, and rats treated with the mabs showed more BDA + axons/section. Data are mean ± SEM (AE12-1, n = 4; AE12-1Y, n = 7; hIgG, n = 4; PBS, n = 4); *p < 0.05, **p < 0.01, one-way ANOVA with Bonferroni’s post-hoc test. (**F**–**G**) In a separate experiment, injured rats were treated with AE12-1Y and were injected with BDA at either 4 or 6 weeks post-SCI, and the (**F**) length and (**G**) number of BDA labeled axons was measured. The average axonal length was significantly greater when BDA was injected at 6 weeks compared to 4 weeks. Data are mean ± SEM (n = 4 per each condition); *p < 0.05, one-way ANOVA, Bonferroni’s post-hoc test.

We examined the effect of inhibiting RGMa with the mabs on axonal regeneration. We quantified the mean number of 5HT+ serotonergic fibers caudal to the lesion site, and found a significantly higher number of 5HT+ fibers in AE12-1 treated rats relative to controls (Fig. 7A,B). Rats injected with AE12-1Y showed a trend towards a higher number of 5HT labeled axons although there was significant variability in the number of 5HT fiber counts in the AE12-1Y groups as reflected in the large standard error. Furthermore, we also examined the effect of RGMa neutralization on descending CST pathways. We injected BDA tracer into the sensorimotor cortex to anterogradely label the CST. Our SCI model of impact-compression injury in which both the dorsal and ventral aspects of the spinal cord are simultaneously compressed results in central cavitation of the gray matter and adjacent white matter, severing all CST axons in the dorsal CST, leaving only a spared rim of subpial white matter. Treatment with AE12-1 or AE12-1Y showed BDA labeled CST fibers caudal to the lesion site 6 weeks after SCI (Fig. 7C). These fibers showed a highly irregular morphology, unlike BDA labeled CST fibers rostral to the lesion or in uninjured rats which are typically long and straight. Both the number and average maximal length of BDA labeled CST fibers was increased in rats injected with either AE12-1 or AE12-1Y (Fig. 7D,E). In contrast, no BDA fibers were observed caudal to the lesion site in control rats. We also quantified the mean axonal length and number of BDA labeled fibers in another group of similarly injured rats treated with AE12-1Y and injected with BDA at 4 or 6 weeks post-SCI. There were more BDA labeled regenerated CST fibers at 6 weeks compared to 4 weeks and BDA labeled fibers were significantly longer at 6 weeks (Fig. 7F,G) suggesting the regeneration of CST axons following treatment with anti-RGMa mabs.

### RGMa mabs attenuate neuropathic pain

**Figure 8:**
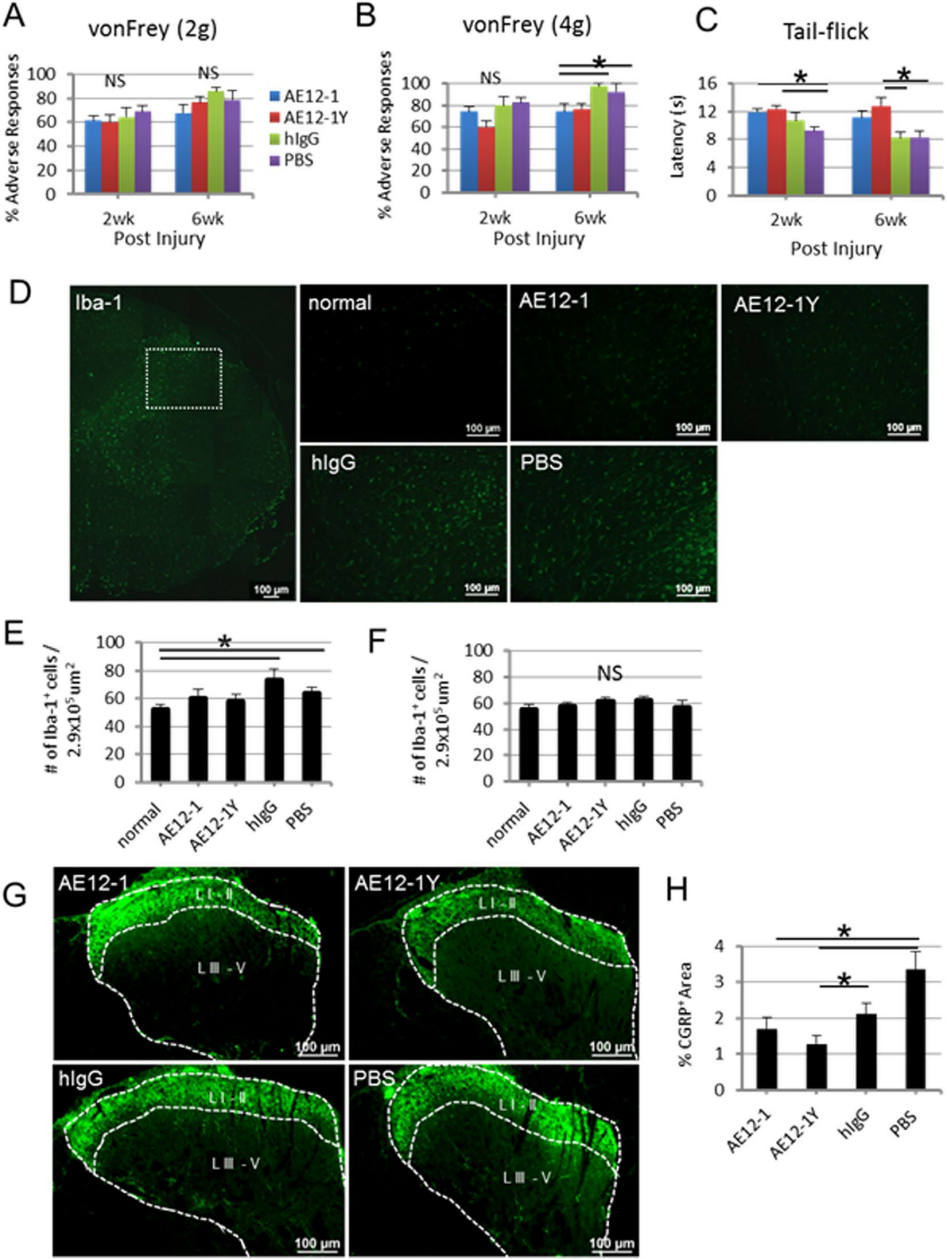
Inhibition of RGMa with mabs attenuates neuropathic pain. Mechanical allodynia at the level of the SCI was assessed with (**A**) 2 g and (**B**) 4 g vonFrey monofilaments. At 6 weeks post-SCI, AE12-1 treated rats showed fewer adverse responses to the 4 g stimulus relative to controls. Data are mean ± SEM (AE12-1, n = 9; AE12-1Y, n = 9; hIgG, n = 7; PBS, n = 7); *p < 0.05, one-way ANOVA with Bonferroni’s post-hoc test. NS, not significant. (**C**) Thermal hyperalgesia was evaluated by the tail flick test by recording the latency to withdrawal of the tail in response to noxious skin heating. At 2 and 6 weeks post-SCI, mab treated rats showed reduced withdrawal to the heat stimulus relative to controls. Data are mean ± SEM (AE12-1, n = 9; AE12-1Y, n = 9; hIgG, n = 7; PBS, n = 7); *p < 0.05, one-way ANOVA, Bonferroni’s post-hoc test. (**D**) Caudal to the lesion at T10, the number of Iba-1 + microglia were counted in the dorsal horn within the boxed region, as shown in the first panel. Representative regions from each group are shown at higher magnification. (**E**) Significantly more Iba-1 + cells were counted in the dorsal horn in injured controls compared to uninjured normal cord. Fewer activated microglia were quantified in the dorsal horns of mab treated injured spinal cords, although this was not statistically significant. Data are mean ± SEM (normal, n = 5; AE12-1, n = 8; AE12-1Y, n = 8; hIgG, n = 7; PBS, n = 7); *p < 0.05, one-way ANOVA with Bonferroni’s post-hoc test. **(F)** In comparison, rostrally at C4 there was no difference in microglial activation in the dorsal horns between groups. NS, not significant. (**G**) CGRP immunoreactivity in dorsal horn at T10 showing fewer CGRP + fibers in laminae III – V (L III - V) in AE12-1 and AE12-1Y treated rats compared to hIgG and PBS controls. (**H**) % CGRP + area was significantly reduced in AE12-1 and AE12-1Y treated rats relative to controls. Data are mean ± SEM (AE12-1, n = 8; AE12-1Y, n = 9; hIgG, n = 7; PBS, n = 5); *p < 0.05, one-way ANOVA with Bonferroni’s post-hoc test.

We assessed the effect of RGMa neutralization on the development of neuropathic pain following SCI. Interestingly, we found that mab treatment reduced at-level mechanical allodynia and thermal hyperalgesia. At 6 weeks post-SCI, rats administered AE12-1 showed significantly fewer adverse responses to the 4 g vonFrey stimulus compared to controls (Fig. 8A,B). Rats treated with either AE12-1 or AE12-1Y showed reduced latency to withdrawal of the tail in response to a heat stimulus compared to controls (Fig. 8C). We then examined the correlation of Iba-1 expression in the spinal cord, but found no significant difference in the percentage area of Iba-1 + staining rostral or caudal immediately adjacent to the lesion site (data not shown). This quantitation included all Iba-1 immunostained cells, including both activated microglia and macrophages. However, Iba-1 + macrophages can easily be distinguished morphologically further rostral or caudal to the lesion site, thus we then specifically examined activated microglia caudal to the lesion at level T10. We quantified Iba-1 + microglia in the spinal cord dorsal horn in cross-sections at T10 caudal to the lesion, as exacerbated microglial activation in the dorsal horn has been associated with the severity of neuropathic pain^24^. In this analysis, only Iba-1 + microglia were counted (Fig. 8D). Although not statistically significant, there were fewer Iba-1 + microglia in the dorsal horn at T10 in mab treated rats relative to controls. Conversely, significantly more Iba-1 + cells were counted in the dorsal horn in injured controls compared to uninjured cord (Fig. 8E). We also quantified Iba-1 + microglia in the dorsal horn of spinal cords at C4, and found no significant difference between groups (Fig. 8F), highlighting the differences seen caudal to the lesion site (Fig. 8E). Furthermore, we examined changes in CGRP expression, shown to be involved in spinal pain mechanisms^25, 26^. Control rats showed significantly greater CGRP + immunoreactive fibers in the dorsal horn compared to mab treated rats (Fig. 8G,H), suggesting a positive effect of RGMa neutralization on the plasticity of pain afferents entering the dorsal horn caudal to the level of injury.

## Discussion

Cell death and the lack of axonal regeneration due to the inhibitory environment of the injured spinal cord are primary causes for poor recovery after SCI. Both intrinsic and extrinsic factors are important for CNS regeneration^27^. Most strategies to repair SCI have focused on either neuroprotection to limit cell death following injury or strategies to promote axonal regeneration. We report here the novel finding that neutralizing the effects of inhibitory RGMa with N-terminal-RGMa-specific human antibodies promotes both neuronal survival and axonal regeneration after impact-compression SCI. Importantly in view of clinical translation, we used human antibodies administered systemically in a clinically relevant model of SCI. Our study demonstrates several key findings: (1) RGMa is upregulated after a clinically relevant impact-compression SCI in rats and also in the injured human spinal cord. (2) Neutralizing RGMa inhibition with clinically relevant human antibodies improved neurobehavioural recovery after acute SCI in multiple functional tests. (3) Inhibiting RGMa promoted perilesional neuronal survival. (4) RGMa inhibition enhanced the plasticity of descending serotonergic pathways and promoted corticospinal tract axonal growth. (5) RGMa inhibition also attenuated neuropathic pain responses, reducing microglial activation and CGRP expression in the dorsal horn caudal to the lesion site. Collectively, these data show the therapeutic benefit of neutralizing inhibitory RGMa with clinically relevant human RGMa-specific antibodies after acute SCI.

RGMa, a potent inhibitor of axonal growth^8^, was previously shown to be upregulated in myelin and the glial scar after dorsal hemisection SCI and ischemic stroke in rats^11, 13, 14^ and in the human brain after traumatic brain injury and focal cerebral ischemia^12^. In the present study, we show for the first time that RGMa is upregulated in multiple cell types after impact-compression SCI in rats and importantly, also in the human spinal cord after traumatic SCI.

Previously we demonstrated a selective interaction between the N-terminal part of RGMa and the immunoglobulin domain in Neogenin that mediates Neogenin recruitment to lipid rafts that is both RGMa and BMP (bone morphogenetic protein)-dependent, and where the receptor acts to transmit guidance signals and control cell death^16^. Using a peptide (4Ig) designed against the immunoglobulin domain prevented RGMa-Neogenin interaction and blocked Neogenin transport into lipid rafts which promoted neuroprotection and axonal regeneration after SCI^16^. In the present study, we used clinically relevant human monoclonal antibodies that are selective for the N-terminal portion of RGMa. We recently showed in biochemical studies that these mabs neutralize inhibitory RGMa by blocking Neogenin function^14^. Thus pleiotropic effects regulated by the mabs reflect the involvement of the Neogenin pathway and the RGMa-BMP pathway. Since the human anti-RGMa mabs show cross-reactivity with rat RGMa, this enabled testing the mabs in a rat SCI model.

In a partial lesion model of thoracic dorsal hemisection injury, intrathecal administration of antibodies generated against the C-terminal portion of RGMa promoted CST axonal growth and functional recovery on the BBB locomotor test^13^. In the present study, we utilized a clinically relevant model of impact-compression SCI that produces pathology similar to human SCI. We show that neutralizing RGMa with systemic injection of the N-RGMa mabs promoted behavioural recovery on several functional tests, including the open field BBB, the horizontal ladderwalk, and computerized CatWalk gait analysis. Treatment with AE12-1 showed earlier functional recovery compared to AE12-1Y where recovery was delayed. The early difference in recovery profiles between these mabs may be due to the longer half-life of AE12-1. This early recovery profile on the BBB test suggests a neuroprotective effect of RGMa inhibition, also supported by the peri-lesional neuronal sparing observed after acute treatment with AE12-1. Following weekly administration of the mabs for 6 weeks, we found antibody concentration still remained elevated in the serum 3 weeks after the last injection. At 6 weeks post-SCI, most of the mab treated rats showed consistent plantar stepping with coordination compared to the control groups. Similarly, the mab treated rats showed fewer footfall errors with a higher success rate on the ladderwalk test, reflecting improved fine motor function, hindlimb weight support, precise paw placement, and better coordinated balance. Moreover, gait analysis demonstrated improved inter-limb coordination in mab treated rats with the hindlimb stride length and swing speed approaching pre-SCI values.

Here we demonstrate multiple mechanisms by which RGMa inhibition with the human mabs facilitates neuroanatomical plasticity of the injured spinal cord. After traumatic brain and spinal cord injury, neurons undergo apoptosis concomitant with axonal degeneration. We found that mab treatment increased neuronal survival after SCI with fewer neurons undergoing apoptosis resulting in a greater number of perilesional neurons remaining after injury. Recently we showed that AE12-1Y mab increased retinal ganglion cell survival in retinal whole mounts following oxygen glucose deprivation, and *in vivo* after ligation of the ophthalmic artery^14^. Furthermore, our data shows inhibition of RGMa enhances the plasticity of descending serotonergic pathways and promotes corticospinal tract axonal growth after SCI. Kyoto *et al*.^28^ showed enhanced CST synapse formation in the cervical region after thoracic SCI and RGMa inhibition. Feng *et al*.^29^ used RNA interference to knock down RGMa via recombinant adenovirus injections after middle cerebral artery occlusion in rats resulting in an increase in CST axonal sprouts towards the ischemic region. Treatment with AE12-1Y mab reduced ischemic damage after middle cerebral artery occlusion resulting in decreased brain infarct size and improved recovery^14^.

Interestingly, we also found that RGMa neutralization attenuated neuropathic pain responses after SCI, with a reduction in mechanical allodynia and thermal hyperalgesia. This was associated with fewer activated microglia and reduced CGRP expression in the dorsal horn caudal to the lesion. Neuropathic pain has been associated with microglial activation and increased CGRP expression in the dorsal horn^24–26^. Moreover, rats treated with RGMa antibody showed increased pressure of hind paws during stance obtained from gait analysis which has previously been shown to correlate with reduced mechanical allodynia^21^. Activated microglia have been shown to inhibit axonal growth through RGMa, and treatment with RGMa-neutralizing antibodies or transfection of RGMa siRNA attenuated the inhibitory effects of microglia on axonal outgrowth^30^. In a focal spinal targeted EAE model in rats, treatment with human anti-N-RGMa mab reduced activated microglia and macrophages promoting regeneration and functional recovery^14^. After SCI, we did not observe fewer macrophages at the lesion site in parasagittal sections from anti-N-RGMa mab injected rats. However, it is conceivable that there was a macrophage shift to an anti-inflammatory phenotype in response to a more permissive microenvironment^31^ although we did not investigate this in the current study. Collectively, these data suggest the therapeutic potential of reducing neuropathic pain responses after SCI with RGMa inhibition and thus warrants further investigation. These data also suggest a new role for the RGMa/Neogenin pathway on neuropathic pain.

In summary, we show therapeutic benefit of neutralizing inhibitory RGMa with clinically relevant novel human RGMa-specific monoclonal antibodies, producing both neuroprotective and regenerative effects in the injured spinal cord and promoting neurological recovery and a reduction in neuropathic pain after SCI.

## Methods

### Ethics statement

All animal procedures were approved by the Animal Care Committee of the Research Institute of the University Health Network (UHN) in accordance with policies established by the Canadian Council on Animal Care. For the harvesting of human spinal cord tissue, approval was obtained from the UHN Research Ethics Board and from the Trillium-Gift of Life Foundation which oversees organ donation in Ontario. In accordance with the guidelines and regulations of the UHN Research Ethics Board, informed consent was obtained for study participation and publication of images from tissue samples. All methods were performed in accordance with the guidelines and regulations of the UHN Research Ethics Board in compliance with the Ontario Personal Health Information Protection Act. All experiments and outcome measures were performed using appropriate blinding.

### Generation of human RGMa monoclonal antibodies

AE12-1 and AE12-1Y are human anti-RGMa monoclonal antibodies (mabs) generated by AbbVie via PROfusion mRNA display using *in vitro* RNA libraries which allow expression and selection of single chain Fv antibodies. Both AE12-1 and AE12-1Y are high affinity, RGMa-selective monoclonal antibodies. These mabs exhibit comparable binding affinity to human, cynomolgous macaque, rat and mouse RGMa. AE12-1Y is a variant of AE12-1 by replacement of an unpaired Cysteine residue (Cys^226^) in light chain complementarity determining region 3. AE12-1 and AE12-1Y, dosed at 4–5 mg/kg intravenously in rats, show a half-life of 6d and 2d in rats, respectively. The anti-RGMa antibody levels in serum used for determining the half-life were measured by Mesoscale Discovery (MSD) assay employing biotinylated human RGMa (0.5 µg/mL) for capture and goat anti-human sulfotag antibody (0.5 µg/mL) for detection as described in the section below. The two mabs do not cross-react with the other two RGM family members RGMb and RGMc, as determined by Surface Plasmon Resonance using BIAcore instrument. Both AE12-1 and AE12-1Y mabs neutralize RGMa activity in functional assays such as chemotaxis, neurite outgrowth, and BMP-responsive reporter gene assays. Human IgG (hIgG) was used as an isotype control for the *in vitro* and *in vivo* studies and was recombinantly produced in CHO cells.

### Assay for measuring antibody levels in serum and CSF

Serum and CSF samples from treated animals were analyzed with a MSD assay employing biotinylated human RGMa (0.5 µg/mL) for capture and goat anti-human sulfotag antibody (0.5 µg/mL) for detection. The samples were analyzed at a 1% final matrix concentration. MSD standard curve fitting and data evaluation was performed using XLfit4 software (Version 4.2.1 Build 16). A calibration curve was plotted from MSD luminescence units versus theoretical standard concentrations. A four-parameter logistic model was used for curve fitting. The regression equation for the calibration curve was then used to back calculate the measured concentrations. The background intensity from animals treated with PBS was subtracted before calculating concentrations from the standard curve. The lower limit of quantitation was 0.007 µg/mL, and the linear range was 5–0.007 µg/mL. Plates were considered valid when at least 2/3 of the quality controls were within 30% of the expected values.

### Western blots

Mouse cortical neurons were lysed in RIPA buffer containing a protease inhibitor cocktail. Samples were denatured at 95 °C for 5 min in a 6X Lamelli buffer (50 mM Tris-HCl [pH6.8], 2% SDS, 10% glycerol, 1% β-mercaptoethanol, 12.5 mM EDTA, and 0.02% bromophenol blue). Samples were loaded on a 10% acrylamide gel in Tris-Glycine running buffer (25 mM Tris, 192 mM Glycine, and 0.1% SDS) and transferred onto a nitro-cellulose membrane. Blots were blocked with 5% nonfat dry milk in PBS, and probed with the anti-RGMa mabs (AE12-1; 1:1000 (1 mg/ml) and AE12-1Y; 1:1000 (1 mg/ml)) and anti-Neogenin (E20; Santa Cruz; 10 mg/ml) at 4 °C overnight. After three washes in PBS containing 0.1% Tween-20, blots were probed with an Odyssey goat anti-mouse secondary antibody (1:4000; Li-COR, Lincoln, Ne, USA) for 2hr at room temperature.

### *In vitro* neuronal immunostaining and neurite outgrowth assay

E18 mouse cortical neurons were plated on poly-L-Lysine (PLL)-coated glass coverslips treated with laminin (Invitrogen; 10 mg/ml) for 24hr and then immunostained with βIII-tubulin (Sigma; 1:500), AE12-1 (1:250), AE12-1Y (1:250) or hIgG (1:250). Embryonic cortical neurons were also stained with F-actin (Molecular Probes; 1:100) and Neogenin (Santa Cruz; 1:200). For the neurite outgrowth assay, E18 mouse cortical neurons were plated on PLL-coated glass coverslips treated with laminin (Invitrogen; 10 mg/ml) and RGMa proteins (5 mg/ml) and incubated for 24hr at 37 °C with control hIgG antibody (1 mg/ml) or anti-RGMa (AE12-1 or AE12-1Y; 1 mg/ml). Cultures were fixed with 4% paraformaldehyde 24hr after plating, and neurons were immunostained with βIII-tubulin (Sigma; 1:500). Experiments were done in duplicates and repeated 3 times. Thus 6 coverslips of neuronal cultures were counted from several areas of the coverslip, and at least 20 neurons were measured per coverslip, as previously described^14, 16^. Fiber length was quantified using Image Pro 5.0.

### Animals

A total of 63 adult female Wistar rats (330–345 g; Charles River, St. Constant, QC, Canada) were used (n = 40 functional/BDA tracing/histological analysis; n = 8 separate BDA tracing experiment; n = 7 RGMa/Neogenin immunostaining; n = 8 TUNEL staining). Efforts were made to minimize the numbers of animals used. Pre-operatively, rats were acclimatized and trained for baseline behavioral assessment. Animals were allocated to treatment and control groups in a randomized manner post-injury.

### Spinal cord injury and injections

Rats were anesthetized by inhalation of 2% isofluorane in combination with a mixture of nitrous oxide and oxygen (1:2, v/v). Under aseptic conditions, a laminectomy was performed at level T9/10. A clip impact-compression injury was made at spinal cord level T8 with a 20 g force for 1 min with a modified aneurysm clip as reported previously^32, 33^. Briefly, and as illustrated in Fig. 1A, the clip is applied extradurally producing a bilateral impact force followed by sustained dorsal-ventral compression, a clinically relevant model of SCI reflecting human pathology^33, 34^. Immediately following SCI, rats were intraspinally injected with either AE12-1, AE12-1Y, hIgG isotype control, or PBS vehicle. There were four groups of rats: 1) AE12-1 (n = 10), AE12-1Y (n = 11), hIgG (n = 11), and PBS (n = 8). The experimental protocol is summarized in Fig. 4A. A total of two injections (2 μg/μL, 3 μL each) were made intraspinally 1 mm rostral and 1 mm caudal to the lesion site and adjacent to the midline vein. All rats received a 20 mg/kg dose of either AE12-1 or AE12-1Y mabs or hIgG or equivalent volume of PBS i.v. via the internal jugular vein, as we previously described^16^. Rats received weekly jugular vein injections for two more weeks and then weekly tail vein injections until 6 weeks post-SCI. In a separate set of rats, SCI was performed as described above and the rats were sacrificed 1 week later and tissue was used for immunostaining for Neogenin and RGMa (n = 4 SCI; n = 3 uninjured). Bladders were expressed three times daily until spontaneous voiding was established. Rats were provided Clavomax (amoxicillin trihydrate/clavulanate potassium) (62.5 mg PO BID for 7 days) in their drinking water to prevent hematuria or urinary tract infection, and rats were housed singly in a temperature-controlled room at 26 °C with a 12hr light/dark cycle.

### BBB and motor subscore

All tests were performed by two independent examiners blinded to treatments. Locomotor function was evaluated using the Basso, Beattie and Bresnahan (BBB) open-field locomotor rating scale^35^ which ranges from a score of 0 indicating no hindlimb movement to 21 indicating normal locomotion as observed in an uninjured rat. Rats were placed individually in an open field with a non-slippery surface and hindlimb motor function including joint movements, stepping ability, coordination, and paw placement were video-recorded. Motor subscores were determined as previously reported^36^ to assess additional measures such as toe clearance, predominant paw position, and absence of instability. A maximal motor subscore of 7 represents normal locomotion.

### Ladderwalk

Fine motor function was assessed with the horizontal ladderwalk apparatus previously described^37^. Rats with a BBB score > 11 were placed on the ladderwalk and 3 runs were recorded which were analyzed in slow motion, and the total number of footfalls per hindlimb was scored for each run and averaged. Injured rats with dragging hindlimbs were scored the maximum footfalls which was 6 footfalls per hindlimb. Uninjured rats had 0 or occasionally 1 footfall per crossing. The relative success rate on the test was calculated as previously described^38^.

### CatWalk

To further elucidate motor function, we performed computerized gait analysis using the CatWalk analysis system^39^ (Noldus Information Technology, Netherlands). Baseline gait assessments were obtained pre-operatively and compared to assessment at 6 weeks post-SCI. Briefly, the system consists of a horizontal glass plate walkway and video capturing equipment placed underneath the walkway and connected to a computer. Digital footprints were acquired with the same experimental settings for each rat and each run was within range for crossing time and speed. A minimum of 3 correct crossings (without pausing or interruption) per animal were obtained and digital footprints were labeled.

### Assessment of mechanical allodynia and thermal hyperalgesia

For tests for neuropathic pain, mechanical allodynia was assessed with vonFrey filaments and the tail flick test was used for thermal hyperalgesia. VonFrey filaments (Stoelting) were used to assess cutaneous sensitivity to normally innocuous mechanical stimulation and were applied to the dermatomes at the level of the SCI as described^40^. A 2 g and 4 g filament was used at each time point. Ten mechanical stimuli to the dorsal surface of the trunk around the level of injury at T8 were made in a clock-wise direction^25^ starting with the 2 g vonFrey filament. Each stimulus was separated by at least a 5 sec interval. Avoidance behavior was defined by one of the following actions: vocalization, flinching, turning away, licking or escaping from the stimulus to another area of the cage. The number of avoidance responses out of 10 was counted to obtain a response percentage. Thermal hyperalgesia was evaluated by the tail flick test by recording the latency to withdrawal of the tail in response to noxious skin heating. An automated analgesia meter (IITC Life Science, Woodland Hills, CA) was used to apply a beam of light to the dorsal surface of the tail at 4 cm from the tip^41^. The time for the rat to flick its tail away from the heat stimulus was recorded, and 3 measurements were made during a 30 min testing session and averaged.

### Tissue processing

Rats were sacrificed and transcardially perfused with 4% paraformaldehyde in 0.1 M PBS, pH 7.4 at 9 weeks after SCI after behavioural assessments and anterograde axonal tracing had been performed. A 1.5 cm segment of tissue encompassing the lesion site at T8 was excised and cryoprotected in 30% sucrose in 0.1 M PBS, embedded in Shandon Cryomatrix (VWR Laboratories) and cryosectioned parasagittally into 20 μm serial sections.

### Anterograde axonal tracing

To visualize axons from the CST, anterograde axonal tracing with BDA was performed 6 weeks after SCI following completion of the functional assessment. Animals (n = 4–7) from each group were randomly selected for BDA injection. As previously described^16^, burr holes were drilled bilaterally to expose the sensorimotor cortex and 1 μl of BDA (10% dissolved in 0.01 M PBS, 10,000 MW; Invitrogen (Thermofisher)) was injected stereotactically at 6 sites^16^ in each sensorimotor cortex at a depth of 1.2 mm. Rat were perfused as described above 3 weeks later and a 1.5 cm segment of tissue encompassing the lesion site was excised and processed as 20 μm parasagittal cryosections. In addition, segments of tissue 5 mm caudal from the brainstem and 5 mm caudal to the lesion site (rostral and caudal segments) were serially cryosectioned transversely. The intensity of BDA staining of the dorsal CST in the rostral segment of each cord was quantified and the ratio was used to normalize the counts of BDA labeled fibers to correct for inter-animal variation in the BDA labeling efficiency. The caudal segment was examined for the presence of any BDA labeled fibers. Sections were processed for BDA as previously described^16^. In each cord, 5 consecutive serial sections containing the maximal area of cavitation were used for quantitation. The length of BDA fibers was measured from the caudal end of the lesion site and averaged. The number of fibers at distances caudal from the lesion site were quantified and binned as follows: <900 μm, 900–1800 μm, 1800–2700 μm. In a separate experiment, rats were injured and injected with AE12-1Y as described above, and then injected with BDA at 4 weeks or at 6 weeks post-SCI (n = 4/group). The number of BDA labeled axons and their length was quantitated as described above.

### Histology and immunostaining

Serial sections (160 μm apart) were immunostained and stained with Luxol Fast Blue and hematoxylin and eosin (LFB/H&E) for general morphology and cavitation analysis. Serial sections for fluorescence immunohistochemistry were rehydrated in 0.1 M PBS, blocked for 1hr, and incubated overnight at 4 °C with the following primary antibodies: NeuN (1:500, Millipore) for neurons, GFAP (1:200, Millipore) for astrocytes, Iba-1 (1:1000, Wako Chemicals) for activated macrophages/microglia, CS56 (1:500, Sigma) for chondroitin sulfate proteoglycan, calcitonin gene-related peptide (CGRP) (1:1000, Millipore) for sensory fibers, 5HT (1:3000, ImmunoStar) for serotonergic fibers, and hIgG (1:500, Millipore) to detect human IgG antibodies (used for staining in Fig. 4). Tissue sections were washed with 0.1 M PBS, incubated with fluorescent-conjugated secondary antibodies for 1hr, washed and then coverslipped with Vectashield mounting medium containing DAPI. Double-labeling was performed as described previously^42^. For RGMa (1:200, Abcam) and Neogenin (1:200, Santa Cruz) immunostaining as shown in Figs 1 and 3E, sections were boiled in 10 mM sodium citrate buffer containing 0.05% Tween20 (pH 6.0) for 10 min at 95–100 °C, blocked with 10% normal goat serum in 0.05% Tween20 for 1hr, incubated overnight with primary antibody diluted in blocking solution, washed and incubated with fluorescent-conjugated secondary antibody for 1hr as above. Species-specific non-immune IgG or omission of primary antibody was used as negative controls. Immunofluorescent staining was examined using a Nikon Eclipse TE 300 microscope and a Zeiss LSM 510 confocal microscope. For immunoperoxidase staining of RGMa and Neogenin in human tissue, sections were dewaxed with xylene and processed through to 100% alcohol. Endogenous peroxidase activity was blocked with 3% H_2_O_2_ in methanol for 30 min. Sections were blocked and incubated with primary antibody as described above, and then incubated with biotinylated anti-mouse secondary antibody (1:500, Vector Laboratories) followed with avidin-biotin-peroxidase complex (Vectastain Elite ABC Kit Standard, Vector Laboratories) for 1hr. Diaminobenzidine (DAB) (Vectastain Elite ABC Kit Standard) was applied as the chromogen. Primary antibody alone and pre-absorption controls with RGMa peptide were run for negative controls, and staining was examined using a Nikon Eclipse TE 300 microscope.

### Quantitative analysis

To quantify spared host neurons, in each cord 5 consecutive sections of the serially sectioned cord containing the maximal area of cavitation were used for quantitation^16, 32^. This technique ensured that equivalent gray matter tissue was analyzed in each animal and also avoided double-counting of cells. The sampled region as outlined in Fig. 6 extended from 2.7 mm rostral to 2.7 mm caudal to the lesion epicentre. Images were captured and cells were manually counted using Nikon NIS Elements BR v.3.1 software. Data are presented as group means of total counts that have not been normalized for the entire cord thickness. For quantification of CSPG immunoreactivity, 3 consecutive serial sections (160 μm apart) in each cord containing the maximal area of cavitation were used for quantification, as we previously described^16, 32^. Using a Nikon Eclipse TE 300 microscope, images encompassing the lesion site were obtained with identical settings and exposure times and stitched with NIS Elements BR software. Images were thresholded using NIH ImageJ software and the fractional area of CSPG immunoreactivity was determined. For quantitation of activated microglia and macrophages, serial sections were stained with Iba-1. In each cord, 3 consecutive serial sections containing the maximal area of cavitation were used. In each section, the sampled regions (2.9 × 10^5^ μm^2^) were 0.45 mm rostral and 0.45 mm caudal to the lesion. Immunostained sections were imaged with identical settings and exposure times as above and thresholded using ImageJ, and the % Iba-1 + area of 3 regions rostral and caudal was averaged for each cord. The % Iba-1 + area included both Iba-1 + activated microglia and macrophages adjacent to the lesion site. At spinal levels C4 and T10, Iba-1 + microglial cells were counted in the superficial dorsal horn (laminae I -III) of serial cross-sections. In each cord, Iba-1 + cells in 3 regions (2.9 × 10^5^ μm^2^) were quantified in the superficial dorsal horn of 3 consecutive serial sections per cord and averaged. To assess CGRP+ primary afferents terminating in the dorsal horn of the spinal cord, CGRP immunoreactivity was quantified in 3 consecutive serial sections at T10 that were imaged with identical settings (2.9 × 10^5^ μm^2^). Using NIH ImageJ software automatic thresholding for each image was performed as described above to obtain the % CGRP+ fractional area. To assess serotonergic fibers, the number of 5HT+ fibers caudal to the lesion site were quantified and binned as follows: <900 μm, 900–1800 μm, 1800–2700 μm. To quantify RGMa immunostaining, 3 regions (2.9 × 10^5^ μm^2^) in 3 serial cross-sections were imaged in each region of interest, thresholded as above using ImageJ, and the % RGMa+ area of the 3 regions were averaged for each of the 3 sections per cord.

### Cavitation analysis

For cavitation analysis, serial sections were stained for LFB/H&E and imaged. The area of cavitation of each section was traced using Nikon NIS Elements v.3.1 software, and the total cavity volume and percentage cavitation was calculated using the Cavalieri method, as previously described^32, 43^.

### Double-label NeuN/TUNEL staining

To evaluate neuronal cell death, a separate experiment was performed where two groups of rats were injured and injected (exactly as described above) with either AE12-1 (n = 5) or PBS (n = 3) and sacrificed 7hr later. To double-label spared neurons with TUNEL, sections were blocked and incubated overnight with mouse anti-NeuN (1:500, Millopore) followed by anti-mouse IgG conjugated to Alexa 488 (1:500; Invitrogen). Sections were washed in PBS and permeabilized by immersing in 0.1% Triton X-100 in 0.1% sodium citrate (v/v) for 2 min and then incubated for 1hr at 37 °C in a humidity chamber in a TUNEL reaction mixture containing a 1:10 ratio of terminal deoxynucleotidyl transferase and fluorescein-conjugated dUTP (*in situ* cell death detection kit; Roche Diagnostics, Laval, QC). Negative control sections were stained as above but in the absence of terminal deoxynucleotidyl transferase. Positive control sections were incubated with 200U/ml DNase 1, grade 1 (Roche Diagnostics) in 50 mM Tris-HCl (pH 7.5) containing 10 mM MgCl_2_ and 1 mg/ml BSA, for 10 min at 37 °C to induce DNA strand breaks prior to labeling procedures. Data shown are representative of three independent experiments. Double-labeled cells were examined using a Zeiss LSM 510 confocal microscope. For each cord, 6 serial cross-sections (160 μm apart) at the epicenter were sampled to avoid double-counting of cells. All NeuN + /TUNEL + double-labeled cells were counted in 6 cross-sections per cord and averaged.

### Statistical analysis

Data are presented as mean ± S.E.M. Functional tests were analyzed by two-way repeated-measures ANOVA comparing groups versus time points followed by post-hoc pairwise multiple comparisons using the Bonferroni method. Statistical differences between multiple groups (vonFrey, tail flick, percentage cavitation, lesion volume, neuronal counts, fiber counts, and % area measurements) were assessed using one-way ANOVA and Bonferroni post-hoc corrections. Differences between two groups (% RGMa immunoreactivity and number of NeuN + /tunel + double-labeled cells) were assessed using a two-tailed Student’s t-test. Differences of p < 0.05 were considered to be statistically significant.

## Electronic supplementary material

Supplementary Figure 1
