# Supplementary material for: RGMa inhibition with human monoclonal antibodies promotes regeneration, plasticity and repair, and attenuates neuropathic pain after spinal cord injury
Source: Sci Rep. 2017 Sep 5;7:10529. doi: 10.1038/s41598-017-10987-7 (PMC5585220; doi:10.1038/s41598-017-10987-7)
Supplement: Supplementary file 1 — Supplementary Figure 1 [file 41598_2017_10987_MOESM1_ESM.pdf]

RGMa inhibition with human monoclonal antibodies promotes regeneration, plasticity and repair, and attenuates neuropathic pain after spinal cord injury

Andrea J. Mothe[1,2], Nardos G. Tassew[1], Alirezha P. Shabanzadeh[1], Romeo Penheiro[1,2], Robin J. Vigouroux[1], Lili Huang[3], Christine Grinnell[3], Yi-Fang Cui[4], Emma Fung[3], Philippe P. Monnier[1,5], Bernhard K. Mueller[3], Charles H. Tator[1,2,6]

[1]Krembil Research Institute, Division of Genetics and Development, Krembil Discovery Tower, Toronto ON, Canada M5T 2S8

[2]Toronto Western Hospital, University Health Network, Toronto ON, Canada M5T 2S8

[3]AbbVie Bioresearch Center, Worcester MA, USA 01605

[4]Neuroscience Research, Research and Development, AbbVie Deutschland GmbH & Co. KG, Knollstrasse, Ludwigshafen, Germany 67061

[5]Department of Ophthalmology and Vision Science, University of Toronto, Toronto ON, Canada M5S 3H6

[6]Department of Surgery, Division of Neurosurgery, University of Toronto, Toronto ON, Canada M5S 3H6

Supplementary Figure 1

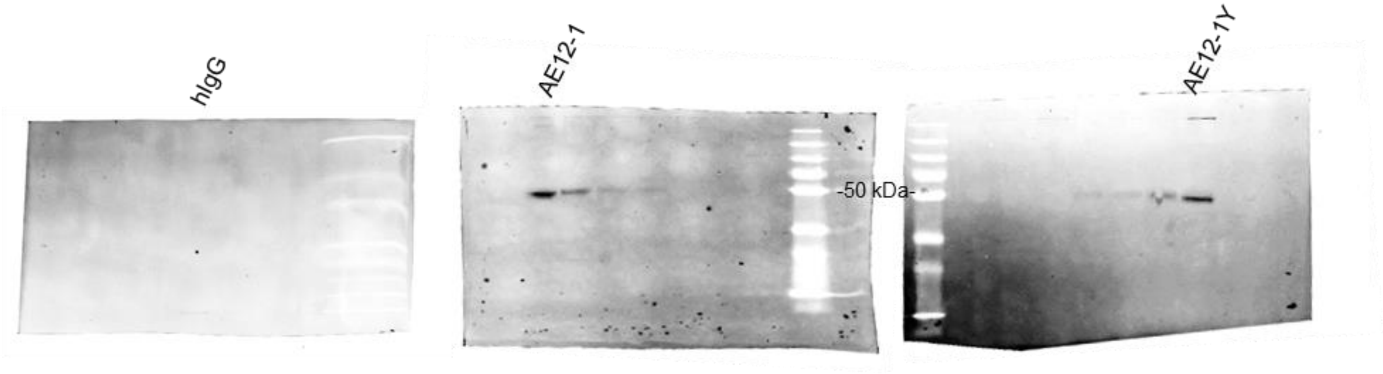

Supplementary Figure 1. Full-length blots showing specificity of the RGMa antibodies. Full-length blots from Figure 3A. Human mabs AE12-1 and AE12-1Y recognized a 50 kDa band on Western blots of mouse cortical neuron lysates. No bands were apparent with the hIgG isotype control antibody. Mouse cortical neurons were lysed and membranes were fractionated using sucrose gradients[20], and blots were probed with hIgG, and the mabs AE12-1 and AE12-1Y, showing specificity of the mabs for RGMa, as we have previously shown[20].
